# In the Shadows of Rarity: A Case Report of Syndromic Cleft Lip and Palate!

**DOI:** 10.7759/cureus.58752

**Published:** 2024-04-22

**Authors:** Pallavi Daigavane, Mrudula Shinde, Priyanka Niranjane, Bhagyashri Chimote

**Affiliations:** 1 Department of Orthodontics and Dentofacial Orthopaedics, Sharad Pawar Dental College and Hospital, Datta Meghe Institute of Higher Education and Research, Wardha, IND; 2 Department of Prosthodontics, Vidarbha Youth Welfare Society Dental College and Hospital, Amravati, IND

**Keywords:** hydrolethalus syndrome, eec syndrome, syndrome, cleft palate, cleft lip

## Abstract

Deviations from normal craniofacial development can result in a range of abnormalities, including cleft lip and/or palate, either as standalone conditions or as components of syndromes with varying clinical characteristics. The ability to distinguish between isolated incidents and syndromes with clefts as one component is integral to achieving accurate diagnosis and therapy.

The following case presentation highlights the importance of comprehensive screening and differential diagnosis in identifying syndromic connections in patients with cleft lip and palate. In this specific case, the patient presented with polydactyly, camptodactyly, and pelvic area abnormalities, indicating a possible syndromic connection with cleft lip and/or palate.

## Introduction

Craniofacial development is one of the most complex events during the embryonic period. Any disturbance in the developmental process during the intra-uterine life may lead to craniofacial abnormalities. Craniofacial abnormalities are considered among the most common birth defects, out of which cleft lip and palate have a higher incidence and prevalence rate. Cleft lip and palate are structural abnormalities between the fourth and 10th week of intrauterine life. This deformity affects one in every 500 or 1000 births all over the world [1]. According to the WHO (2004), a child is born with a cleft every two minutes somewhere in the world. The etiology of this malformation is still unknown, but both genetic and environmental factors may be responsible for any craniofacial abnormalities [2]. Environmental factors such as poor nutrition, smoking tobacco, viral infection, and medicinal drugs are considered to be probable risk factors for the development of cleft lip and/or palate [3].

Cleft lip and palate are classified into syndromic and non-syndromic clefts. The syndromic form of cleft is associated with more than 200 conditions and is seen in 5-7% of total cases of cleft. This form is usually accompanied by the presence of other craniofacial anomalies with a specific malformation pattern [4]. The etiology of the syndromic cleft is usually chromosomal abnormalities; however, environmental factors may also be responsible. Cohen (1978) stated that 154 syndromes were associated with cleft lip and/or palate [5]. However, most of the syndromes are associated with cleft palate followed by syndromes associated with cleft lip and palate [6]. He also stated that most of these syndromes have monogenic etiologic factors followed by chromosomal factors. Syndromes such as Appelt syndrome caused by the COL4A5 Gene and inherited in an X-linked pattern, Gorlin syndrome caused by chromosome 9q22.3 microdeletion, Goldenhar syndrome caused by chromosome 14q32 and microdeletion at 22q11, Van der Woude syndrome caused by the deletion of chromosome 1q32-q41, Apert syndrome caused by a mutation in gene FGFR2, cleidocranial dysplasia caused by a mutation in the RunX2 gene, Larsen syndrome caused by a mutation in Filamin B, Treacher Collins syndrome caused by a deletion in the region of 4p15, etc. are usually seen associated with cleft lip and/or palate.

Identifying the associated syndrome with cleft helps us assess and evaluate the problems and risk factors posed to the child. Also, knowledge regarding the various anomalies with the syndrome helps us in optimum treatment time and planning for the child. This article presents a case report showcasing some of the systemic manifestations that may present in cases of syndromic cleft lip and palate.

## Case presentation

**Figure 1:**
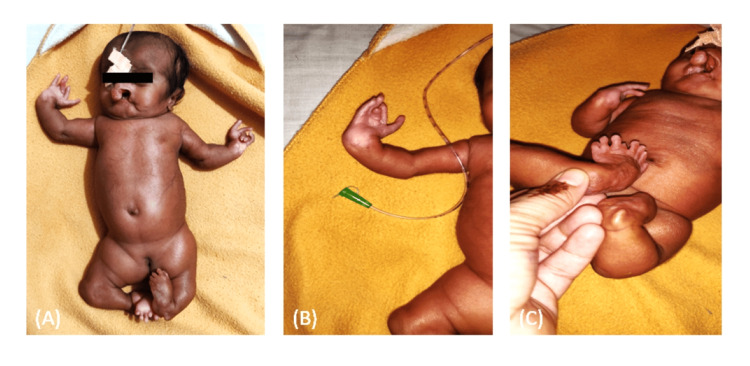
(A) Congenital depression on the forehead of the skull, (B) polydactyly of hands, (C) camptodactyly of hands and feet

A 47-day-old female infant was reported in the Department of Orthodontics and Dentofacial Orthopaedics, Datta Meghe Institute of Higher Education and Research, Wardha, Maharashtra, India, with complete bilateral cleft lip and palate and congenital depression on the forehead of the skull (Figure 1A) [7]. The infant was seen with polydactyly of hands and feet, along with camptodactyly of hands and feet (Figure 1B) and (Figure 1C).

**Figure 2:**
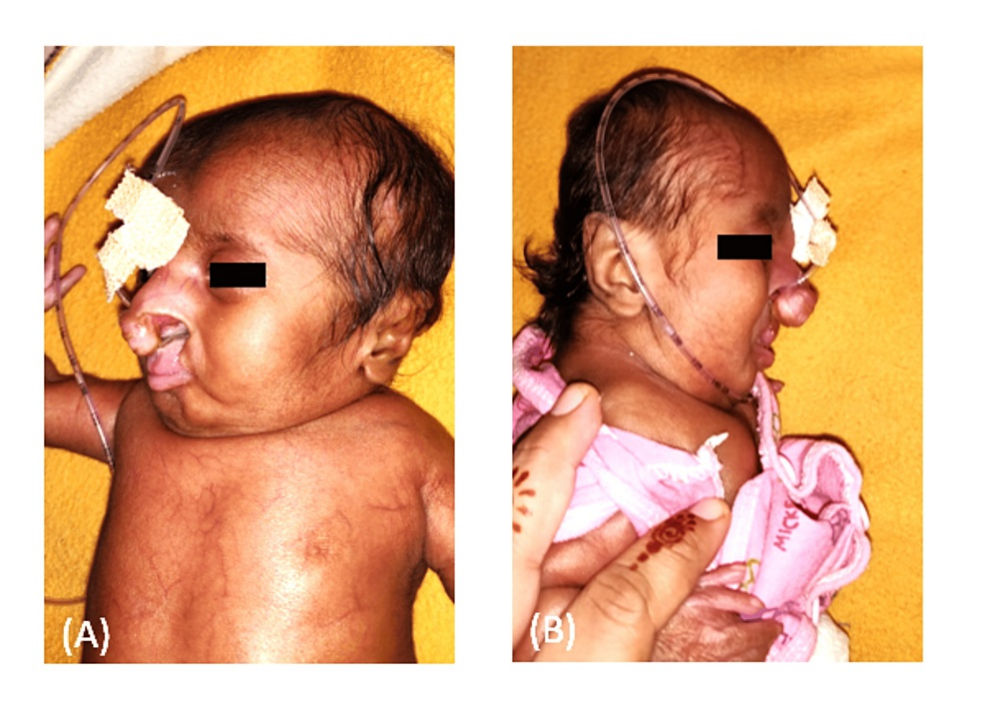
(A) Prominent prolabium, (B) collapsed nasal structure

On extra-oral examination, prominent prolabium and collapsed nasal structure were also evident (Figure 2A) and (Figure 2B), a unique feature with bilateral cleft lip and palate. Abnormal or incomplete formation of the pelvic region was noted (Figure 1A).

**Figure 3:**
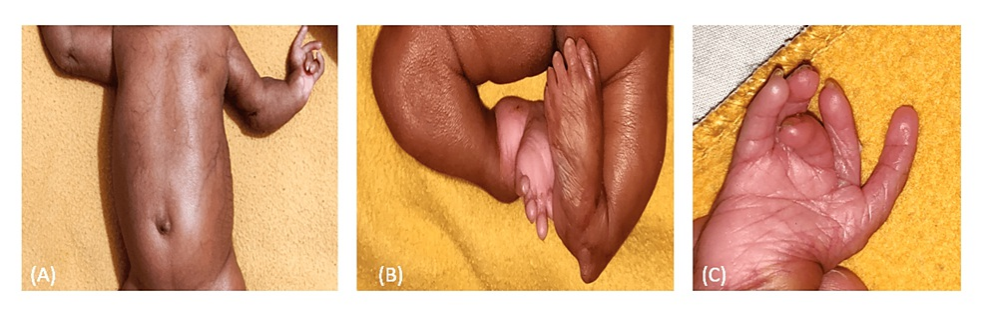
(A) Abdominal telangiectasia, (B) club foot, (C) triphalangeal thumb

The infant also had transient abdominal telangiectasia (also known as venous geography) (Figure 3A) [8], for which echocardiography and a regular ultrasound of the abdominal organs were recommended. Club foot was a typical feature seen in the infant (Figure 3B), which is documented to be associated with one in every 1000 infants with cleft lip and palate. Triphalangeal thumb was present on both the limbs (Figure 3C) [9].

A thorough examination was suggested to identify the syndrome and the risk factors involved with it. Further investigations such as MRI, radiography, and complete blood examination were indicated. The infant was referred to the Smile Train unit of the Department for the Presurgical Infant Orthopedics. Taking into consideration the anomalies associated with the cleft lip and palate, the infant was diagnosed with a syndromic cleft lip and palate with various features associated. She was further referred to the Genetic Laboratory at the Research House of the Datta Meghe Institute of Higher Education and Research for further investigations and specific diagnosis.

## Discussion

It is established that deviations in the development of normal craniofacial complex can lead to abnormalities in this region of which one is cleft lip and/or palate. Cleft lip and/or palate can occur as isolated entities, but many times they are associated with various syndromes that present with different clinical features. The clinician must be able to differentiate between these isolated events and any syndromes of which clefts may be just a component.

In the above-mentioned case, the patient reported polydactyly, camptodactyly, and a defect in the formation of the pelvic region, so it was most likely associated with a syndrome. Based on the clinical features, the differential diagnosis was suggestive of the term "Hydrolethalus syndrome," introduced by Salonen et al. in 1981, which describes a condition characterized by significant central nervous system malformations, hydrocephalus, micrognathia, cleft lip/palate, lung hypoplasia, clubfoot, and polydactyly. It can be differentiated from Meckel syndrome by the absence of polycystic kidneys in affected patients [10]. As described by Rüdiger et al. in 1970 and Pinheiro and Freire-Maia in 1994, ectrodactyly-ectodermal dysplasia clefting syndrome manifests with three distinctive features. These include ectrodactyly and syndactyly affecting the hands and feet, cleft lip with or without cleft palate (potentially leading to speech challenges), and anomalies in several ectodermal structures, including the skin and hair [11,12]. Further investigations were suggestive of a final diagnosis. However, the following are some of the other syndromes that this case may represent: Goldenhar syndrome, Gordon syndrome, and Meckel-Gruber syndrome.

Goldenhar syndrome, also known as oculo-auriculo-vertebral spectrum, consists of a classical triad of abnormal development of first and second branchial arches, ocular dermoid, and vertebral anomalies. Principal characteristics of this syndrome are cleft lip and/or palate, rib anomalies, polydactyly or clinodactyly of hands and feet, congenital heart disease, mental retardation, and growth hormone deficiencies. Certain principle features were seen in this case; however, some other features were not seen which may be due to the young age and a lack in the investigation that had been done [13].

Gordon syndrome is an autosomal dominant condition that is characterized primarily by the presence of camptodactyly of hands and feet, cleft palate, and clubbed digits of hands and feet. Camptodactyly is characterized by flexion contracture of the proximal interphalangeal joint, i.e., the presence of bent digits of hands and feet. Other features which may or may not be present in this condition are scoliosis, ptosis, and mental retardation [14]. The primary features of Gordon syndrome were seen in the above case; however, a severe form of the cleft defect was seen in the patient. A thorough examination is necessary to obtain a proper diagnosis.

Meckel-Gruber syndrome is a rare autosomal recessive disorder with characteristic features such as occipital encephalocele, polydactyly, and dysplastic cystic kidneys. Other abnormalities associated with it are cleft lip and/or palate, genital anomalies, and central nervous system (CNS) malformation. It cannot be concluded conclusively, however, whether the patient presented with Meckel-Gruber syndrome as she presented with only two features [6]. Further investigations are required which will confirm the presence or absence of the mentioned syndrome. Roberts-SC phocomelia syndrome is another rare and inherited syndrome that presents with micrognathia, microcephaly, scanty hair growth, delayed growth, and in some cases may also present with cleft lip and/or palate. A few features of this syndrome were found to present in the above case; however, further confirmatory findings need to be made to diagnose the patient with this condition.

## Conclusions

We as clinicians need to conduct the appropriate investigations, not just to reach a justifiable diagnosis, but also to rule out other similar syndromes and conditions. This will ensure that the patient receives optimum care and that the proper therapy can be provided. In this case report, the patient presented with few clinical features that are present in a number of syndromes. Only after complete clinical investigations have been performed can the final diagnosis be given. It is essential that in such cases we thoroughly inspect all the signs and symptoms pertaining to each condition and come to a justifiable diagnosis.
